# Serum CD4 Is Associated with the Infiltration of CD4^+^T Cells in the Tumor Microenvironment of Gastric Cancer

**DOI:** 10.1155/2021/6539702

**Published:** 2021-02-28

**Authors:** Qi You, Tianyi Fang, Xin Yin, Yimin Wang, Yongheng Yang, Lei Zhang, Yingwei Xue

**Affiliations:** ^1^Department of Gastrointestinal Surgery, Harbin Medical University Cancer Hospital, Harbin Medical University, Harbin, Heilongjiang 150086, China; ^2^Department of Pathology, Harbin Medical University, Harbin, Heilongjiang 150001, China

## Abstract

Serum CD4, CD8, and CD19 are markers of systemic inflammation. However, there is little evidence on the influence of inflammation on the tumor microenvironment and the prognostic indicators of gastric cancer (GC). In this study, two hundred and eight patients who underwent radical gastrectomy for GC were included. Preoperative peripheral blood samples were used to analyze Serum CD4, CD8, and CD19. The optimal cutoff levels for CD4, CD8, and CD19 were defined by receiver operating characteristic curve analysis (CD4 = 38.85%, CD8 = 14.35%, and CD19 = 7.40%). The areas with specific CD4^+^T cells, CD8^+^T cells, and CD19^+^B cells within the tumor microenvironment were measured in paraffin sections by immunohistochemistry and analyzed by Image-Pro Plus. 94 patients had low CD4, and 124 patients had high CD4 levels. 31 patients had low CD8, and 187 patients had high CD8 levels. 64 patients had low CD19, and 154 patients had high CD19 levels. Infiltration of CD4^+^T cells was associated with serum CD4 (*P* < 0.001). Serum CD4 and CD19 and the infiltration of CD4^+^T cells, CD8^+^T cells, and CD19^+^B cells were significant in predicting the prognosis of GC. Low CD4 level, infiltration of CD8^+^T cells, and high infiltration of CD4^+^T cells and CD19^+^B cells were correlated with worse overall survival in multivariate analysis. Collectively, our results provide evidence that serum CD4 is associated with the infiltration of CD4^+^T cells in the tumor microenvironment, which indicates the prognostic value of systemic inflammation in GC.

## 1. Introduction

According to the latest global cancer report, more than 70% of new cases of gastric cancer (GC) and deaths are from developing countries, causing a social burden that cannot be ignored [1, 2]. Recent years, immunotherapy is proving to be an effective therapeutic method in a variety of cancers, but researchers have realized that the effects of immunotherapy often vary greatly from individual to individual [3, 4]. In patients with pathological response of tumor regression or no response, the 5-year survival rate differs by more than 50% [5–7]. Therefore, in addition to providing standardized treatment for patients, it is also necessary for clinicians to identify sensitive, easily available, and low-cost markers to provide a basis for individualized treatment and prediction of prognosis in GC patients.

Chronic local inflammation has been shown to play an important role in tumorigenesis and progression. Inflammatory cells are also considered to be an important part of the tumor microenvironment [8, 9]. Pathologists have noted that tumor-associated neutrophils, tumor-associated macrophages, and tumor-associated lymphocytes exist in the microenvironment of various malignancies [10]. From 2013 to 2018, Galon et al. [11, 12] first proposed the incorporation of TNM-I (TNM-immune) into the tumor staging criteria, and the Union for International Cancer Control (UICC) included the degree of infiltration of immune cells in tumor microenvironment into the pathological staging of colon cancer [13]. The predictive effect of tumor-associated immune cells on the prognosis of patients has been a hot research topic worldwide. Recent researches have shown that tumor-infiltrating lymphocytes can be used as predictive biomarkers for immunotherapy sensitivity in patients with liver cancer [14, 15]. These studies have indicated that inflammatory cells in the tumor microenvironment can not only classify patients into pathological stages but also play a valuable predictive role in the response to cancer immunotherapy.

The systemic chronic inflammatory response is also thought to be associated with tumor prognosis. These predictors include the neutrophil-lymphocyte ratio (NLR), platelet-lymphocyte ratio (PLR), C-reactive protein, and procalcitonin. Many studies have shown that these markers are independent factors in the prognosis of patients with lung cancer [16], liver cancer [17], pancreatic cancer [18], and colon cancer [19]. In 2017, Choi et al. [20] first reported that the NLR and PLR were associated with the density of immune cells in the tumor microenvironment, which leads to prognostic values of systemic inflammation in gastric cancer. However, the main limitation of this kind of researches, which are aimed at reflecting the systemic inflammatory response by using this ratio, was that it could not accurately reflect the changing characteristics of immune cell subsets [21]. This difference in subsets such as CD4, CD8, and CD19 cells affects the sensitivity and effectiveness of immunotherapy more precisely, but there are still no other comprehensive researches to clarify this phenomenon.

At present, the infiltration of immune cells in the tumor microenvironment can only be evaluated using pathological sections and microscopy. But due to the high heterogeneity of GC, there is a great variety of randomness in the choice of pathological section and observation field, which result in difficulties in terms of clinical application. A variety [11] of cytokines and inflammatory cells are currently known to migrate from the peripheral blood into the tumor microenvironment mainly through the systemic circulation [22]. Thus, whether peripheral blood with a controllable detection level can reflect the infiltration of immune cells in the tumor microenvironment is worth further study.

In the present study, we investigated the prognostic value of CD4^+^T cells, CD8^+^T cells, and CD19^+^B cells in the peripheral blood by flow cytometry, as well as in the tumor microenvironment by immunohistochemistry of GC, and evaluated their correlations in different locations in order to determine their complicated interactions.

## 2. Materials and Methods

### 2.1. Patient Characteristics

A total of 218 patients with GC were randomly selected from August 2014 to June 2015 in the Department of Gastrointestinal Surgery, Harbin Medical University Cancer Hospital. The diagnosis of GC was based on the pathological report following gastroscopy. All patients received D2 lymphadenectomy, and the specimens were pathologically examined. The included patients underwent abdominal ultrasound, stomach CT/MRI, chest film, ECG and other examinations, and some patients underwent PET-CT scanning. The patients were staged according to the American Joint Committee on Cancer (AJCC)/UICC 8th edition staging system. The exclusion criteria were as follows: (a) preoperative radiotherapy or chemotherapy, (b) distant metastasis, (c) recurrent GC, or the presence of another tumor, (d) antiplatelet therapy within the previous three months, (e) septicemia or severe systemic infection (f) patients with blood disorders or multiple myeloma, and (g) medical records were incomplete. All patients were followed up after discharge. The median follow-up time was 42 months (range 0-60 months). The medical records were included in the GC Information Management System version 1.2 (Copyright No. 2013SR087424) of Harbin Medical University Cancer Hospital. This study was approved by the Ethics Committee of Harbin Medical University.

### 2.2. Laboratory Examinations

Peripheral blood samples were collected from patients within 3 days before surgery for routine laboratory examination to determine the leukocyte count, neutrophil count, lymphocyte count, and levels of serum CD4, CD8, and CD19. According to the receiver operating characteristic curve, an optimal threshold value was defined in order to divide the samples into two groups (high group and low group) in relation to the inflammatory markers.

### 2.3. Density of Immune Cells in the Tumor Microenvironment

Immune cells in the tumor microenvironment were analyzed by immunohistochemistry using paraffin sections. Tissue samples from surgical specimens were fixed in 10% formalin for 48 hours and then embedded in paraffin. Paraffin sections from the 218 GC patients were dewaxed in xylene and ethanol. After cleaning in distilled water, the paraffin sections were pretreated with citrate buffer at pH 8.0 (CD4, CD8, and CD19) for 3 min at 120°C in a pressure cooker, and endogenous peroxidase was inhibited with 3% H_2_O_2_ in PBS for 10 min. Nonspecific actions in the sections were also blocked with goat serum (BOSTER, Pleasanton, CA, USA) for 1 hour at room temperature. The sections were then incubated with the primary antibody overnight at 4°C and for 30 min with the secondary antibody at 37°C. The primary antibodies used were CD4 (ab183685, 1 : 1000, Abcam, Cambridge, MA, USA), CD8 (ab4055, 1 : 100, Abcam, Cambridge, MA, USA), and CD19 (ab134114, 1 : 300, Abcam, Cambridge, MA, USA). The secondary antibody was goat anti-rabbit IgG (CD4, CD8, and CD19). The chromogenic reaction was performed via diaminobenzidine (DAB) staining, and the staining intensity was measured using Image-Pro Plus version 6.2 software (Media Cybernetics, Rockville, MD, USA).

All specimens were reviewed by two independent blinded pathologists based on the percentage of positively stained cells. In order to minimize the heterogeneity of immune cell distribution, a series of optimization experiments were performed to reduce subjective factors. The pathologists carefully examined hematoxylin and eosin (H&E) staining of multiple wax blocks from the same patient sample before the experiment without knowing the identity of the patients. The most representative wax block covering multiple heterogeneous regions was selected for the experiment using the same criteria. In order to reduce the effect of heterogeneity, image information was collected from lymphocyte-enriched regions, interstitial regions, and tumor-cell-enriched regions, respectively, and the average area was estimated by relative percentage staining and intensity staining. Three representative images with a magnification of ×200 were collected, and the area of immunostaining in each image was measured using Image-Pro Plus version 6.2 software. In addition, all enrolled patients did not receive preoperative chemotherapy and radiotherapy, thus eliminating the effects of chemotherapy and radiotherapy on tumor cells and immune cells.

### 2.4. Statistical Analysis

Differences in serum CD4, CD8, and CD19 levels were assessed by the Chi-square test. Correlation coefficients were calculated by the *t*-test or Pearson correlation analysis to assess the correlation between serum inflammatory markers and the percentage of immune cells in the tumor microenvironment. Overall survival (OS) was determined from the date of surgery to the date of the last follow-up or death of any cause. The Kaplan-Meier method was used to calculate the survival rate, and the log-rank test was used for statistical analysis. Multivariate analysis of prognostic factors was performed using the Cox proportional hazard model. A *P* value less than 0.05 was considered statistically significant. Statistical analysis was performed using SPSS 22.0 (Chicago, IL, USA).

## 3. Results

### 3.1. Patient Characteristics

Of the 218 patients, 166 (76.1%) were men, and 52(23.9%) were women. There were 38 elderly patients (over 70 years), accounting for 17.4%. All patients underwent major gastrectomy. According to the Borrmann classification, 40.8% of the patients were of Borrmann type III (40.8%) and 17.4% were of Borrmann type I. 39.4% of the patients showed vascular infiltration, and 62.4% of the patients showed nerve invasion. According to the 8th edition of the AJCC staging system, stage I patients accounted for 17.9%, stage II patients for 26.6%, stage III patients for 49.1%, and stage IV patients for 6.4%. With regard to lymph node involvement, lymph nodes were negative in 66 (30.3%) patients, and the lymph node ratio (ratio of the number of invaded lymph nodes to the number of lymph nodes examined) was between 85.0% and 39.0%. Forty-four (20.2%) patients had a lymph node ratio between 0.3 and 0.6, and 23 (10.6%) patients had a lymph node ratio greater than 0.6. With regard to tumor location, entire stomach GC accounted for 7.3%, upper third GC for 8.3%, middle third GC for 18.8%, and lower third GC for 65.6% (Table 1).

### 3.2. Relationship between Clinical Pathology and Blood Inflammatory Markers

Median serum CD4 was 40.1% (range 19.4%-66.2%), and we defined 38.85% as the cut-off value. Ninety-four patients were in the low CD4 group, and 124 patients were in the high CD4 group. Median serum CD8 was 22.57% (range 4.4%-56.5%), and we defined 14.35% as the cut-off value. Thirty-one patients were in the low CD8 group, and 187 patients were in the high CD8 group. Median serum CD19 was 9.7%, and we defined 7.40% as the cut-off value. Sixty-four patients were in the low CD19 group and 154 patients were in the high CD19 group.

Serum CD4 was found to be associated with the Borrmann classification and lymph node metastasis, while serum CD4 and CD19 were associated with age. The lower levels of CD4 (*P* = 0.024) and CD19 (*P* = 0.020) were more common in patients younger than 70 years, and higher Borrmann type and lymph node metastasis were related to increased serum CD4 (*P* = 0.041, *P* = 0.026; Table 2).

### 3.3. Survival Analysis in relation to Serum Inflammatory Markers and Immune Cell Infiltration of the Tumor Microenvironment

**Figure 1:**
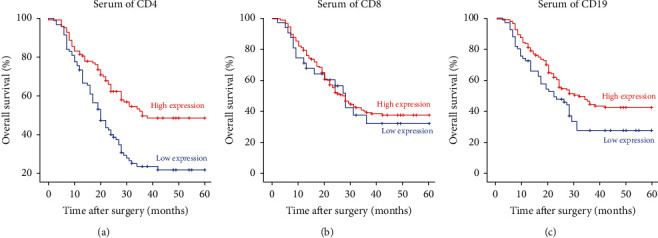
Overall survival of patients with GC stratified by the systemic inflammatory markers CD4, CD8, and CD19.

By analyzing the level of serum inflammatory markers and the prognosis of patients, we found that the 5-year OS rate of patients with high serum CD4 was better than that of patients with low serum CD4 (Figure 1(a)) and was 48.5% and 21.8%, respectively. There was no significant difference in 5-year OS rate between patients with high serum CD8 and low serum CD8 (Figure 1(b)). The 5-year OS rate of patients with high serum CD19 was better than that of patients with low serum CD19 (Figure 1(c)) and was 41.4% and 26.6%, respectively.

**Figure 2:**
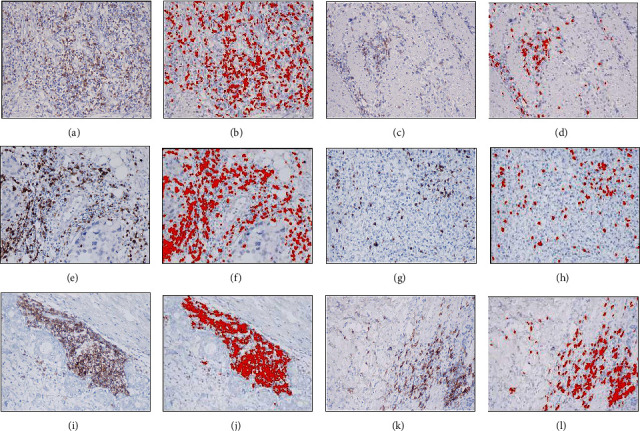
Immunohistochemistry of CD4^+^T, CD8^+^T, and CD19^+^B cells in GC paraffin sections. The positive part was marked red by Image-Pro Plus. (a) High infiltration of CD4^+^T cells. (c) Low infiltration of CD4^+^T cells. (e) High infiltration of CD8^+^T cells. (g) Low infiltration of CD8^+^T cells. (i) High infiltration of CD19^+^B cells. (k) Low infiltration of CD19^+^B cells.

**Figure 3:**
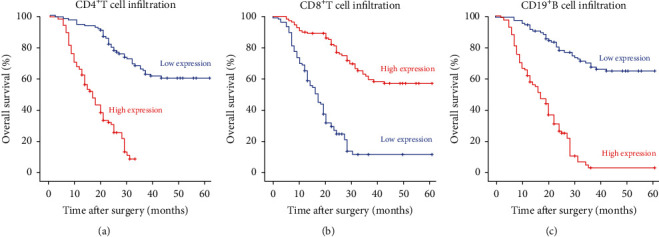
Overall survival of patients with GC stratified by infiltration of immune cells in the tumor microenvironment.

Figure 2 shows the representative immunohistochemistry of immune cells (Figure 2(a): high infiltration of CD4^+^T cells; Figure 2(c): low infiltration of CD4^+^T cells; Figure 2(e): high infiltration of CD8^+^T cells; Figure 2(g): low infiltration of CD8^+^T cells; Figure 2(i): high infiltration of CD19^+^B cells; Figure 2(k): low infiltration of CD19^+^B cells). Image-Pro Plus can digitalize the pathological imaging information, produce direct images, make corresponding statistical analysis, and avoid effect of human factor in the traditional assessment methods of pathological section. We analyzed the percentage of the positive part (red) in the total area and defined the median as the cut-off value (Figures 2(b), 2(d), 2(f), 2(h), 2(j), and 2(l)). By analyzing immune cell infiltration in the tumor microenvironment and prognosis, we found that the 5-year OS rate in the group with high CD4^+^T cell infiltration was lower than that in the group with low CD4^+^T cell infiltration (Figure 3(a)) and was 9.1% and 60.4%, respectively. The 5-year OS rate in the group with high CD8^+^T cell infiltration was better than that in the group with low CD8^+^T cell infiltration (Figure 3(b)) and was 57.5% and 12.4%, respectively. The 5-year OS rate in the group with high CD19^+^B cell infiltration was lower than that in the group with low CD19^+^B cell infiltration (Figure 3(c)) and was 3.7% and 65.1%, respectively. Compared with the level of immune cells in the peripheral blood and the infiltration of immune cells in the tumor microenvironment, CD4^+^T cells and CD19^+^B cells showed the opposite trend in peripheral blood and in the tumor microenvironment.

Considering that there is a significant correlation between the immune cells, we performed univariate analysis and multivariate analysis with Cox's proportional hazards model separately. Each kind of circulating CD4^+^T cell, CD8^+^T cell, and CD19^+^B cell was included as an independent prognostic factor in the multivariate model for OS. Univariate analysis showed that serum CD4 (HR 1.814, 95% CI 1.255-2.622, *P* = 0.002) and serum CD19 (HR 0.653, 95% CI 0.451-0.944, *P* = 0.023) were associated with prognosis. Multivariate analysis showed that serum CD4 (HR 0.597, 95% CI 0.409-0.873, *P* = 0.008) and serum CD19 (HR 0.548, 95% CI 0.365-0.821, *P* = 0.004) were independent prognostic factors in the OS multivariate model (Table 3).

### 3.4. Relationship between the Levels of Serum Inflammation and Tumor Infiltrating Immune Cells

**Figure 4:**
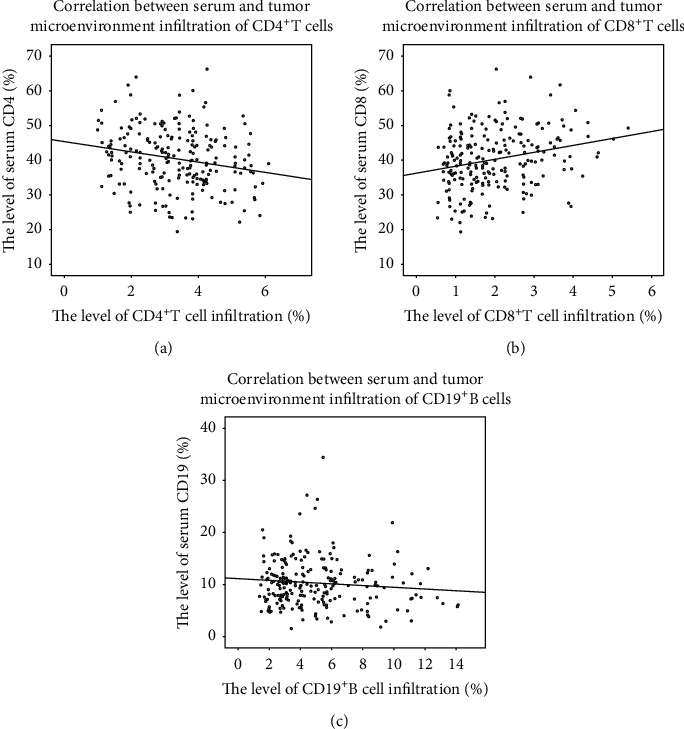
Correlations between serum CD4, CD8, and CD19 and infiltration of immune cells in the tumor microenvironment.

The percentage of infiltrated area of CD4^+^T cells, CD8^+^T cells, and CD19^+^B cells in the tumor microenvironment was 3.42% ± 1.08%, 1.76% ± 0.96%, and 4.16% ± 2.20%, respectively. The percentage of CD4^+^T cell infiltrated area was 3.11% ± 1.21% and 3.68% ± 1.16% in the high serum CD4 group and the low serum CD4 group, respectively. The percentage of CD8^+^T cell infiltrated area was 1.99% ± 1.02% and 1.93% ± 0.98% in the high serum CD8 group and the low serum CD8 group, respectively. The percentage of infiltrated area of CD19^+^B cell in the high serum CD19 group and low serum CD19 group was 5.03% ± 3.43% and 4.07% ± 2.47%, respectively. The Pearson correlation coefficient analysis showed that serum CD4 levels were correlated with CD4^+^T cell infiltration in the tumor microenvironment (correlation coefficient = −0.209, *P* < 0.005, Figure 4(a)), but CD8 and CD19 did not show this trend (correlation coefficient = −0.088, *P* = 0.197, Figure 4(b); correlation coefficient = −0.101, *P* = 0.138, Figure 4(c)).

## 4. Discussion

GC is a common malignant digestive system tumor with a high incidence and short survival period in China [23]. Almost 50% of new GC cases worldwide were from China and more than 60% were at an advanced stage [24]. Surgery is also the key treatment in advanced GC, but the effect is always limited. More than half of patients with advanced GC who only received surgery relapsed within months to two years [25]. Therefore, the surgical treatment without adjuvant therapy cannot significantly improve survival probability. In recent years, with continued understanding of the molecular biological characteristics of GC, immunotherapy, as a new tumor treatment option, has achieved significant results in the treatment of melanoma [26] and hematological tumors [27] and has shown great potential in the clinical treatment of advanced GC [28]. Immunotherapy mainly includes natural immunotherapy, tumor vaccine therapy, adoptive immunotherapy, and immune checkpoint inhibitor therapy [29].

In a study of pembrolizumab in the treatment of PD-L1-positive patients with advanced GC, 53% of patients had tumor regression, 22% achieved partial remission imaging, and the safety was better than second-line chemotherapy [30]. However, because of the individual differences in drug sensitivity and side effects during immunotherapy for GC, there are no objective parameters in clinical practice to help the preliminary screening of sensitive patients. In 2014, The Cancer Genome Atlas (TCGA) proposed a new molecular typing of GC for the first time, including Epstein-Barr virus (EBV) positive, Microsatellite Instability (MSI), Genomically Stable (GS), and Chromosomal Instability (CIN), which is conducive to the screening of targeted drugs for individualized treatment of GC [13]. However, to date, there are still no reliable biomarkers to predict the efficacy of these immunotherapies and the long-term survival probability.

The present study found that serum CD4 and CD19 as biomarkers of systemic inflammation had better prognostic value for GC. It was also found that the level of inflammatory cell infiltration in the tumor microenvironment, including CD4^+^T cells, CD8^+^T cells, and CD19^+^B cells, was of certain significance in predicting the prognosis of GC. In particular, the change in serum CD4 was correlated with the infiltration of CD4^+^T cells in the tumor microenvironment. This indicated that the analysis of the peripheral blood can be used to assess immune cell infiltration in the tumor microenvironment. This information may be valuable in selecting patients with GC who may benefit from immunotherapy.

Previous studies have shown that immune cell infiltration in the tumor microenvironment has a prognostic value in many types of cancer, which is similar to our findings [31]. However, few studies have evaluated the association among prognosis, the tumor microenvironment, and systemic inflammatory response. We pointed out that a low serum CD4 level or increased CD4^+^T cell infiltration in the GC microenvironment can predict a poor prognosis, and this may be related to the function of CD4^+^T cells in the peripheral blood. Circulating CD4^+^T cells can target cancer cell surface antigens and activate peripheral blood CD8^+^T cells to enter the tumor microenvironment, which can activate the function of killing cancer cells [32, 33]. Moreover, our research also found that increased CD8^+^T cell infiltration in the tumor microenvironment often indicates a good prognosis, which may explain this phenomenon.

The main function of peripheral blood CD19^+^B cells is to secrete immunoglobulins (including IgG, IgA, IgM, IgD, and IgE) in order to exert humoral immune functions [34]. In the peripheral blood of patients with GC, B cells can regulate their own functions and release inflammatory factors to inhibit T cells and natural killer T cells so as to play an antitumor role [35]. The reason why CD19^+^B cells in the tumor microenvironment indicate a poor prognosis, which is not consistent with the function of peripheral blood, can be explained by the following mechanism. In 2019, Gu et al. [36] demonstrated that tumor cells can “domesticate” B cells to promote lymph node metastasis in breast cancer. Humoral immunity mediated by tumor-educated B cells and their derived antibodies plays an important role in lymph node premetastatic niche formation. The ability of B cells to secrete antibodies (especially IgG) was significantly enhanced, and the pathogenic IgG could specifically target glycosylated membrane protein HSPA4, which could directly promote tumor metastasis by activating the HSPA4-binding protein ITGB5 and the downstream Src/NF-*κ*B pathway. This may also explain why B cells in the peripheral blood of GC patients have different functions from those infiltrating the tumor microenvironment. GC cells can “domesticate” peripheral blood B cells and humoral immunity contributes to the progression of cancers. When B cells enter the tumor microenvironment, they interact with tumor cells to promote cancer development. However, this conjecture requires further functional tests for verification.

In addition, this study also found that serum CD4 and CD19 were significantly different in GC patients of different ages, and the levels of these two immune cells were higher in patients aged over 70 years. It can be seen that with increasing age, the human immune system changes markedly. There is an effect of aging on cells of the immune system, on soluble molecules, on lymphoid organs, and the initiation of immune responses [37]. Moreover, some individuals arrive to advanced ages without any major health problems, called healthy aging. The immune system dysfunction seems to be somehow mitigated in this population [38]. This indicates that clinical immunotherapy requires evaluation of the immune status of patients in combination with their age, which will be helpful in screening out drug-sensitive patients.

Based on our findings, there is a correlation between serum inflammatory markers and immune cell infiltration in the tumor microenvironment. Thus, we propose that serum CD4 can be used to evaluate immune status in the tumor microenvironment. These markers can be easily obtained from the peripheral blood without invasive tissue biopsy. This type of inflammatory marker in the peripheral blood is also expected to evaluate the efficacy of immunotherapy in patients with GC.

However, this study has some limitations. First, different subtypes of T cells and B cells were not randomly distributed. Tumor-infiltrating immune cells appear in different parts of the tumor microenvironment, such as in the center of the tumor, at the invasive margin of tumoral nests, and in adjacent tertiary lymphoid structures, and may have different functions [11]. Therefore, the role of different subtypes and different infiltration areas of immune cells requires further investigations. When performing peripheral blood tests, the detection of CD4 and CD8 double positive immune cells is unavoidable, and this may have had an impact on our conclusions. Second, it may not be possible to completely rule out systemic inflammatory reactions not caused by cancer, such as those caused by pneumonia, gastritis, *Helicobacter pylori* infection, and autoimmune diseases.

## 5. Conclusions

In conclusion, our research shows that the systemic inflammatory markers, serum CD4 and CD19 and tumor infiltration of CD4^+^T cells, CD8^+^T cells, and CD19^+^B cells can predict the prognosis of GC patients. In addition, the level of serum CD4 is related to the infiltration of CD4^+^T cells in the tumor microenvironment. This systemic inflammatory marker in the peripheral blood is expected to predict the prognosis of GC patients and sensitivity to immunotherapy.

## Figures and Tables

**Table 1:** Patient characteristics.

Variables	Number of patients	Percentage
Sex		
Male	166	76.1
Female	52	23.9
Age (years)^a^		
<70	180	82.6
≥70	38	17.4
Borrmann classification^b^		
Type I	38	17.4
Type II	45	20.6
Type III	89	40.8
Type IV	46	21.1
Vascular invasion		
Absent	132	60.6
Present	86	39.4
Neural invasion		
Absent	82	37.6
Present	136	62.4
TNM stage^C^		
I	39	17.9
II	58	26.6
III	107	49.1
IV	14	6.4
Lymph node ratio (involved/examined lymph nodes)		
0	66	30.3
>0 to ≤0.3	85	39.0
>0.3 to ≤0.6	44	20.2
>0.6	23	10.6
WHO classification		
Well to moderately differentiated	96	44.0
Poorly differentiated	55	25.2
Signet ring cell	54	24.8
Mucinous	13	6.0
Tumor location		
Lower third	143	65.6
Middle third	41	18.8
Upper third	18	8.3
Entire stomach	16	7.3

^a^Median age: 61 years, range 30–83 years. ^b^Borrmann type I: polyp type; Borrmann type II: local infiltration type; Borrmann type III: ulcer type; Borrmann type IV: diffusely infiltrative type. ^C^Based on the 8th edition of the *AJCC Cancer Staging Manual* of the American Joint Committee on Cancer.

**Table 2:** Associations between clinicopathologic variables and systemic inflammatory markers in GC.

Variables	CD4		*P*	CD8		*P*	CD19		*P*
	Low (*n* = 94)	High (*n* = 124)		Low (*n* = 31)	High (*n* = 187)		Low (*n* = 64)	High (*n* = 154)	
Sex			0.559			0.858			0.137
Male	69	97		24	142		53	113	
Female	24	28		7	45		11	41	
Age (years)			0.024			0.110			0.020
<70	72	111		23	160		48	135	
≥70	21	14		8	27		16	19	
Borrmann classification^a^			0.041			0.280			0.246
I	11	27		9	29		12	26	
II	15	30		5	40		14	31	
III	47	42		10	79		30	59	
IV	20	26		7	39		8	38	
Vascular invasion			0.071			0.839			0.960
Absent	49	81		19	111		38	92	
Present	44	44		12	76		26	62	
Neural invasion			0.091			0.592			0.742
Absent	29	53		13	69		23	59	
Present	64	72		18	118		41	95	
TNM stage^b^			0.174			0.071			0.941
I	11	28		4	35		13	26	
II	25	33		10	48		16	42	
III	49	58		12	95		31	76	
IV	8	6		5	9		4	10	
Lymph node ratio (involved/examined lymph nodes)			0.026			0.873			0.147
0	23	45		9	59		22	46	
>0 to ≤0.3	36	49		14	71		18	67	
>0.3 to ≤0.6	19	25		5	39		15	29	
>0.6	15	6		3	18		9	12	
WHO classification			0.895			0.127			0.827
Well to moderately differentiated	43	53		17	79		28	68	
Poorly differentiated	24	31		3	52		17	38	
Signet ring cell	21	33		10	44		14	40	
Mucinous	5	8		1	12		5	8	
Tumor location			0.778			0.071			0.204
Lower third	7	11		2	16		37	106	
Middle third	18	23		2	39		14	27	
Upper third	63	80		22	121		5	13	
Entire stomach	5	11		5	11		8	8	

^a^Borrmann type I: polyp type; Borrmann type II: local infiltration type; Borrmann type III: ulcer type; Borrmann type IV: diffusely infiltrative type. ^b^Based on the 8th edition of the *AJCC Cancer Staging Manual* of the American Joint Committee on Cancer.

**Table 3:** Univariate and multivariate analyses of independent risk factors for death of patients with GC.

Variables	Univariate analysis^c^			Multivariate analysis^d^		
	HR	95% CI	*P*	HR	95% CI	*P*
Univariate and multivariate analyses with the circulating CD4^+^T cells						
Sex			0.285	—	—	—
Male	1					
Female	0.792	0.517-1.215				
Age (years)			0.159	—	—	—
<70	1					
≥70	1.378	0.882-2.154				
Tumor location			0.007			0.662
U	1			1		
M	1.083	0.522-2.246		0.768	0.360-1.637	
L	0.837	0.432-1.622		0.751	0.383-1.473	
LMU	2.306	1.022-5.204		1.033	0.436-2.451	
Borrmann type^a^			<0.001			0.056
I	1			1		
II	1.009	0.477-2.133		0.504	0.229-1.107	
III	2.711	1.454-5.055		1.042	0.524-2.072	
IV	2.947	1.531-5.673		1.161	0.572-2.358	
WHO classification			0.083			
Well to moderately differentiated	1			1		
Poorly differentiated	1.491	0.950-2.338		1.784	1.050-3030	
Signet ring cell	1.584	1.017-2.468		1.565	0.961-2.551	
Mucinous	2.003	1.006-3.989		0.975	0.428-2.220	
TNM stage^b^			<0.001			<0.001
I	1			1		
II	7.116	2.152-23.526		6.457	1.883-22.135	
III	14.823	4.678-46.969		12.624	3.636-43.828	
III	26.953	7.479-97.131		22.266	5.663-87.553	
Vascular infiltration			<0.001			0.750
Absent	1			1		
Present	2.065	1.446-2.947		1.069	0.709-1.611	
Neural invasion			<0.001			0.811
Absent	1			1		
Present	2.330	1.534-3.538		0.943	0.584-1.524	
Serum CD4			0.002			0.008
Low	1			1		
High	1.814	1.255-2.622		0.597	0.409-0.873	
Univariate and multivariate analyses with the circulating CD8^+^T cells						
Sex			0.285	—	—	—
Male	1					
Female	0.792	0.517-1.215				
Age (years)			0.159	—	—	—
<70	1					
≥70	1.378	0.882-2.154				
Tumor location			0.007			0.765
U	1			1		
M	1.083	0.522-2.246		0.788	0.369-1.682	
L	0.837	0.432-1.622		0.792	0.403-1.555	
LMU	2.306	1.022-5.204		1.036	0.435-2.466	
Borrmann type^a^			<0.001			0.014
I	1			1		
II	1.009	0.477-2.133		0.502	0.229-1.104	
III	2.711	1.454-5.055		1.160	0.589-2.284	
IV	2.947	1.531-5.673		1.356	0.677-2.715	
WHO classification			0.083			0.669
Well to moderately differentiated	1			1		
Poorly differentiated	1.491	0.950-2.338		1.313	0.784-2.197	
Signet ring cell	1.584	1.017-2.468		1.253	0.764-2.056	
Mucinous	2.003	1.006-3.989		1.438	0.668-3.093	
TNM stage^b^			<0.001			<0.001
I	1			1		
II	7.116	2.152-23.526		6.551	1.909-22.487	
III	14.823	4.678-46.969		13.398	3.847-46.659	
III	26.953	7.479-97.131		23.754	6.022-93.704	
Vascular infiltration			<0.001			0.672
Absent	1			1		
Present	2.065	1.446-2.947		1.094	0.723-1.654	
Neural invasion			<0.001			0.739
Absent	1			1		
Present	2.330	1.534-3.538		0.922	0.571-1.487	
Serum CD8			0.667	—	—	—
Low	1					
High	0.896	0.543-1.477				
Univariate and multivariate analyses with the circulating CD19^+^B cells						
Sex			0.285	—	—	—
Male	1					
Female	0.792	0.517-1.215				
Age (years)			0.159	—	—	—
<70	1					
≥70	1.378	0.882-2.154				
Tumor location			0.007			0.832
U	1			1		
M	1.083	0.522-2.246		0.722	0.340-1.534	
L	0.837	0.432-1.622		0.734	0.373-1.443	
LMU	2.306	1.022-5.204		0.738	0.302-1.803	
Borrmann type^a^			<0.001			0.006
I	1			1		
II	1.009	0.477-2.133		0.468	0.212-1.032	
III	2.711	1.454-5.055		1.062	0.539-2.092	
IV	2.947	1.531-5.673		1.458	0.727-2.920	
WHO classification			0.083			
Well to moderately differentiated	1					
Poorly differentiated	1.491	0.950-2.338				
Signet ring cell	1.584	1.017-2.468				
Mucinous	2.003	1.006-3.989				
TNM stage^b^			<0.001			<0.001
I	1			1		
II	7.116	2.152-23.526		6.824	1.997-23.318	
III	14.823	4.678-46.969		14.851	4.264-51.726	
III	26.953	7.479-97.131		30.290	7.609-120.574	
Vascular infiltration			<0.001			0.740
Absent	1			1		
Present	2.065	1.446-2.947		.073	0.707-1.630	
Neural invasion			<0.001			0.700
Absent	1			1		
Present	2.330	1.534-3.538		0.910	0.565-1.468	
Serum CD19			0.023			0.004
Low	1			1		
High	0.653	0.451-0.944		0.548	0.365-0.821	

^a^Borrmann type I: polyp type; Borrmann type II: local infiltration type; Borrmann type III: ulcer type; Borrmann type IV: diffusely infiltrative type. ^b^Based on the 8th edition of the *AJCC Cancer Staging Manual* of the American Joint Committee on Cancer. ^c^Log rank test was used for univariate analysis. ^d^Cox regression model was used for multivariate analysis.

## Data Availability

The datasets used in this study are available from the corresponding author on reasonable request. More information can also be obtained from the Gastric Cancer Information Management System v1.2 of Harbin Medical University Cancer Hospital (Copyright No. 2013SR087424, http://www.sgihmu.com/).
